# Supplementary material for: Recombination of ecologically and evolutionarily significant loci maintains genetic cohesion in the Pseudomonas syringae species complex
Source: Genome Biol. 2019 Jan 3;20:3. doi: 10.1186/s13059-018-1606-y (PMC6317194; doi:10.1186/s13059-018-1606-y)
Supplement: Supplementary file 1 — Figure S1. Assembly statistics for all genomes used in this study. Figure S2. Effects of core genome frequency cut-off on the size of the soft-core genome. Figure S3. Rarefaction curves for the core genome of each phylogroup, as estimated using PanGP. Figure S4. Rarefaction curves for the pan genome of each phylogroup, as estimated using PanGP. Figure S5. Number of genomes in which each ortholog family resides. Figure S6. Evolutionary rates for different strain pairs in the Pseudomonas syringae species complex. Figure S7. Genetic architecture of the different type III secretion systems found in the Pseudomonas syringae species complex. Figure S8. Distribution of the different Pseudomonas syringae complex type III secretion systems on the core-genome phylogenetic tree. Figure S9. Maximum likelihood phylogenetic tree of the HrcV structural protein found in all Pseudomonas syringae complex type III secretion systems. Figure S10. Phylogenetic analysis of Pseudomonas syringae strains based on type III secreted effector (T3SE) content (A) and exchangeable effector locus (EEL) content (B). Figure S11. Phylogenetic distribution of eight major phytotoxins produced by Pseudomonas syringae strains. Figure S12. Comparison of the results of four different recombi4nation analysis pipelines. Figure S13. Frequency of horizontal gene transfer into the Pseudomonas syringae species complex. Figure S14. Relationships between inter-phylogroup recombination, virulence, and positive selection for genes in primary Pseudomonas syringae phylogroups based on chisquared proportions tests. Table S1. Gene Ontology annotations assigned to the novel candidate type III effectors in the Pseudomonas syringae species complex. Table S2. Gene Ontology (GO) terms significantly associated with the virulence related ortholog families in the Pseudomonas syringae species complex (FDR p value < 0.05). Table S3. Results of chi-squared equality of proportions tests for relationships between inter-phylogroup rec [file 13059_2018_1606_MOESM1_ESM.pdf]

Supplemental Material For

Recombination of ecologically and evolutionarily significant loci maintains genetic cohesion in the *Pseudomonas syringae* species complex

Marcus M. Dillon, Shalabh Thakur, Renan N.D. Almeida, Pauline W. Wang, Bevan S. Weir, and David S. Guttman

Table of Contents

1. SUPPLEMENTAL FIGURES 3
1.1. Fig. S1 3
1.2. Fig. S2 4
1.3. Fig. S3 5
1.4. Fig. S4 6
1.5. Fig. S5 7
1.6. Fig. S6 8
1.7. Fig. S7 9
1.8. Fig. S8 10
1.9. Fig. S9 11
1.10 Fig. S10 .... 12, 13
1.11 Fig. S11 14
1.12 Fig. S12 15
1.13 Fig. S13 16
1.14 Fig. S14 17
2. SUPPLEMENTAL TABLES 18
2.1. Table S1 18
2.2. Table S2 19
2.3. Table S3 20

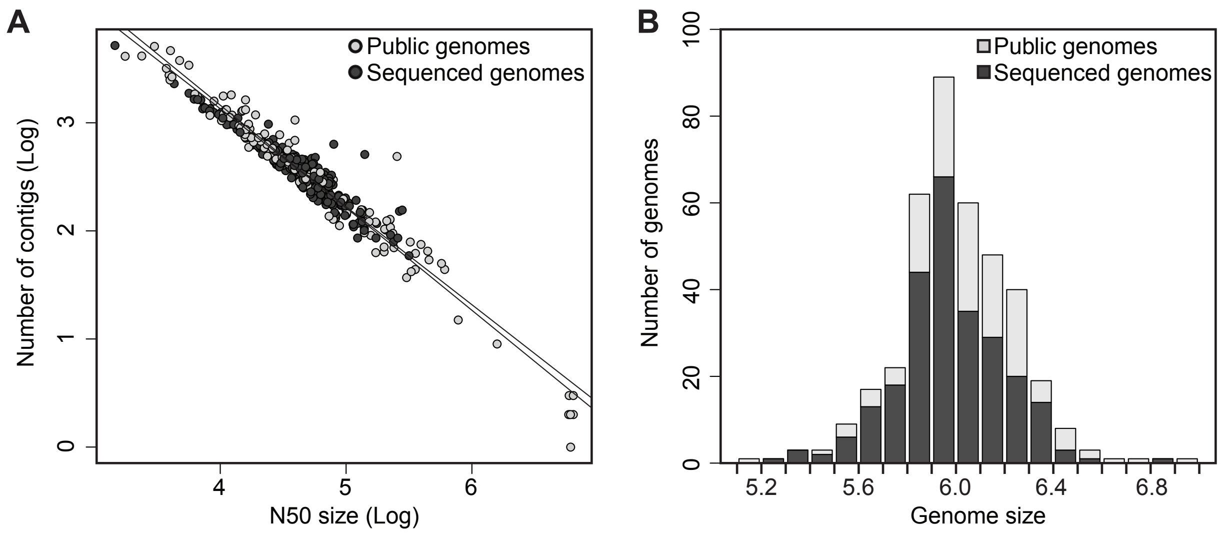

Fig. S1: Assembly statistics for all genomes used in this study. The number of contigs (A), the N50 values (A), and the genome sizes (B) of the genomes that we sequenced and assembled in this study are comparted to those that were downloaded from public databases.

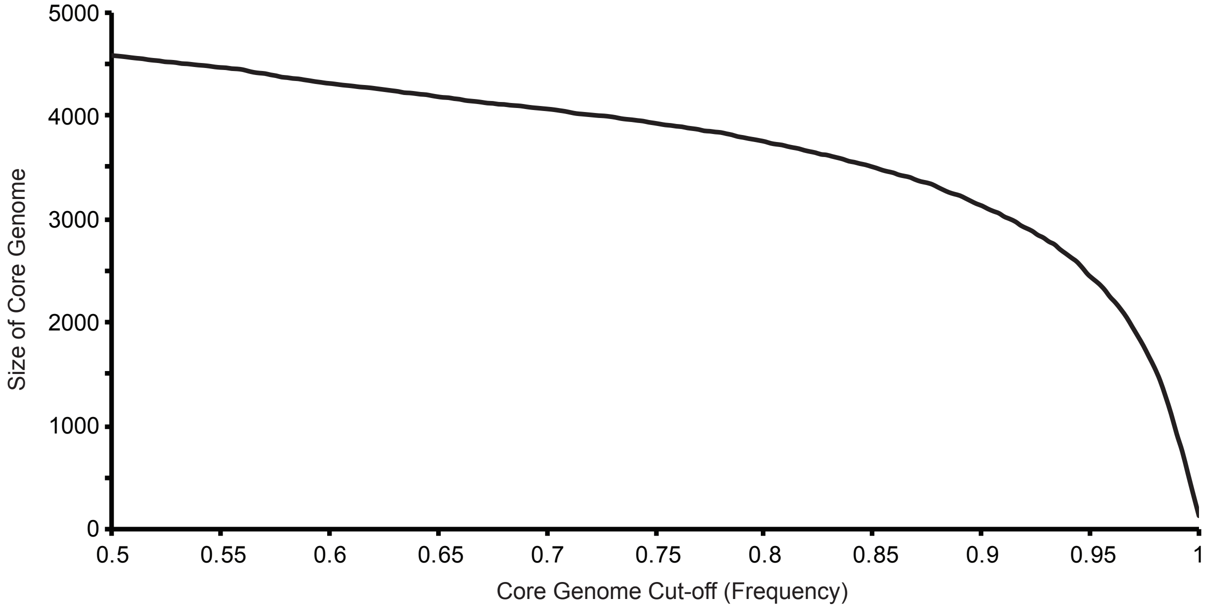

Fig. S2: Effects of core genome frequency cut-off on the size of the soft core genome. The size of the core genome drops off dramatically when greater than 95% of genomes are required for an ortholog family to be considered part of the core genome.

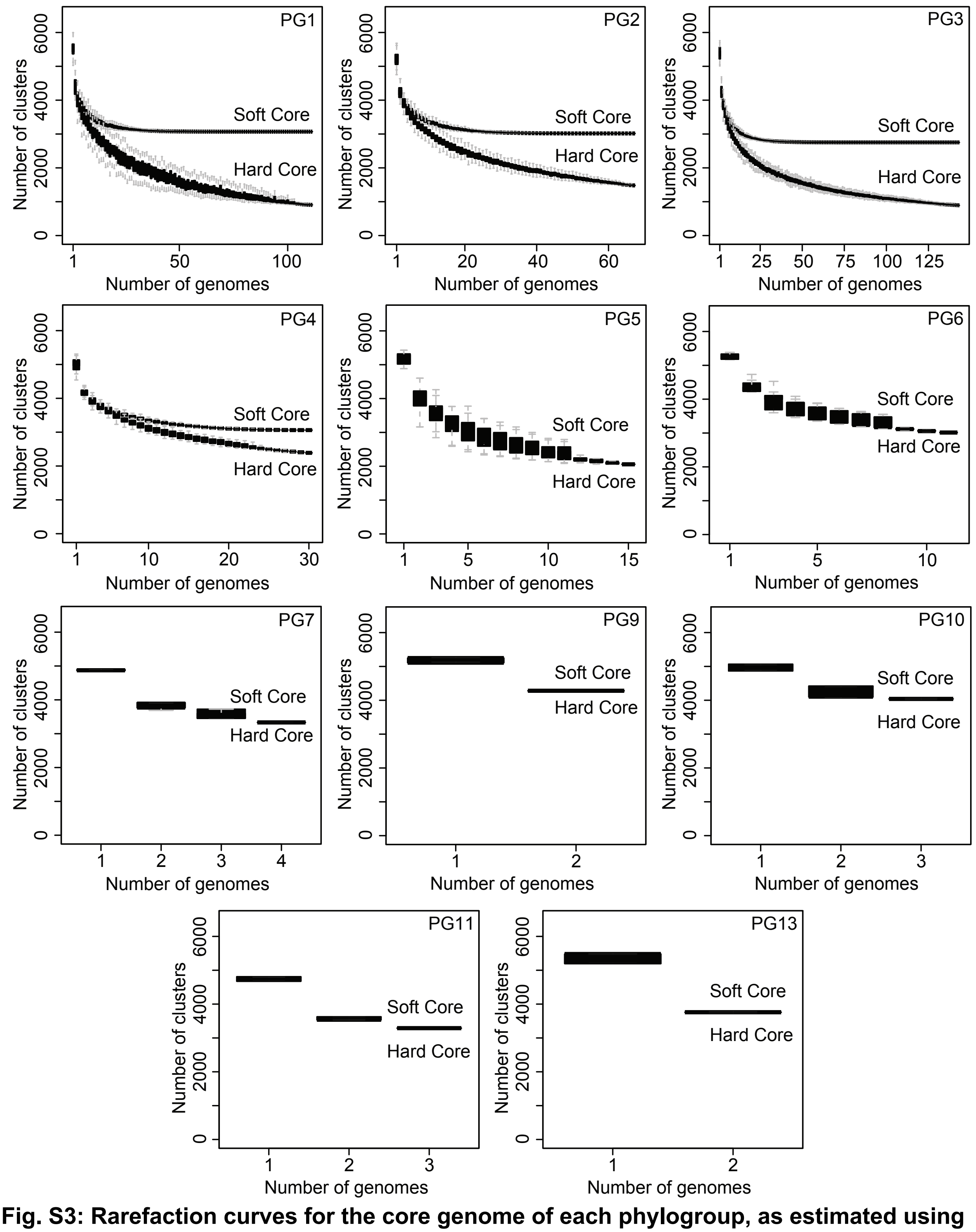

Fig. S3: Rarefaction curves for the core genome of each phylogroup, as estimated using PanGP. Families present in 95% of strains are considered part of the soft core genome and families present in 100% of strains are considered part of the hard core genome.

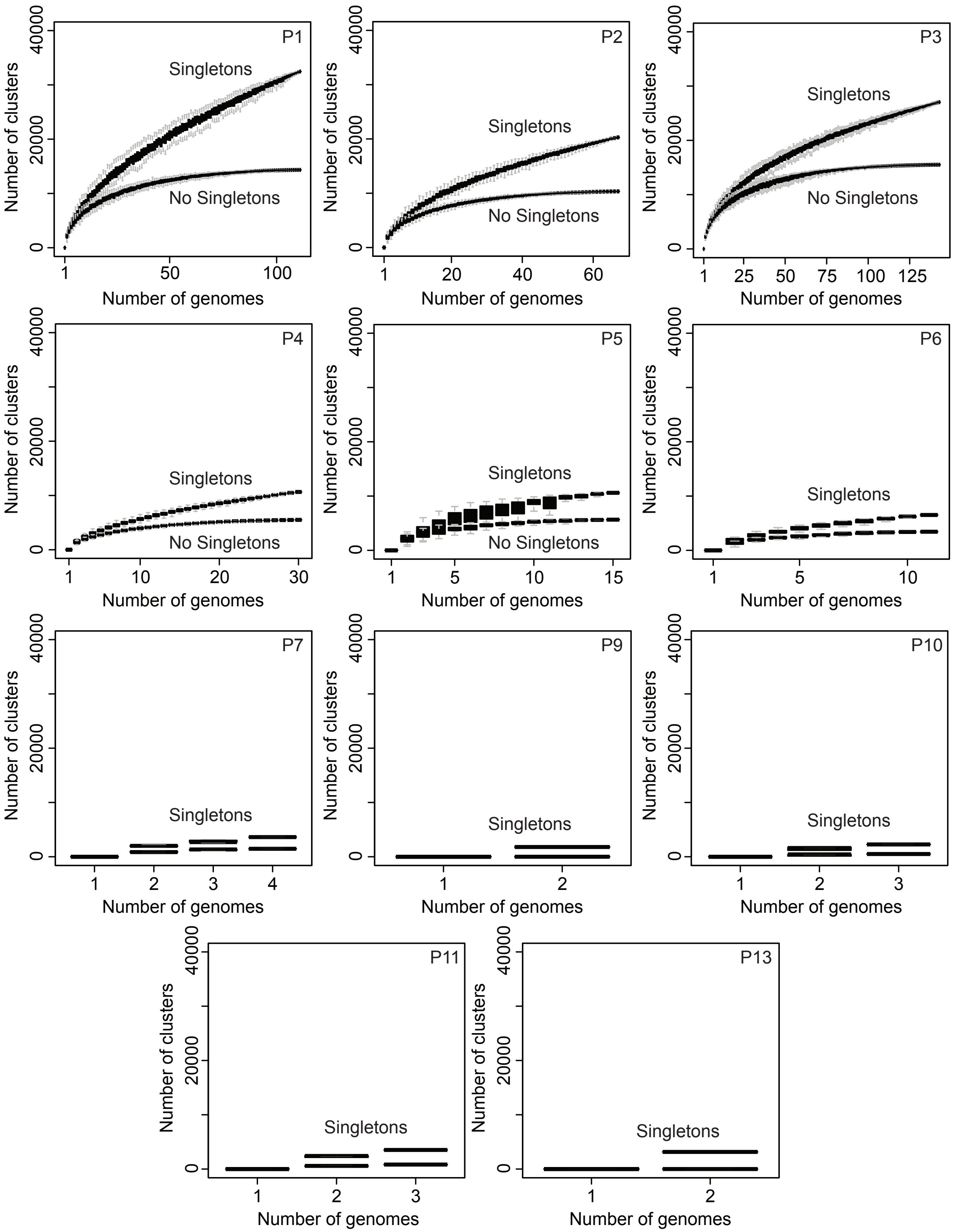

Fig. S4: Rarefaction curves for the pan genome of each phylogroup, as estimated using PanGP. The analysis was performed both when singletons were included (including singletons) and when singletons were excluded (excluding singletons).

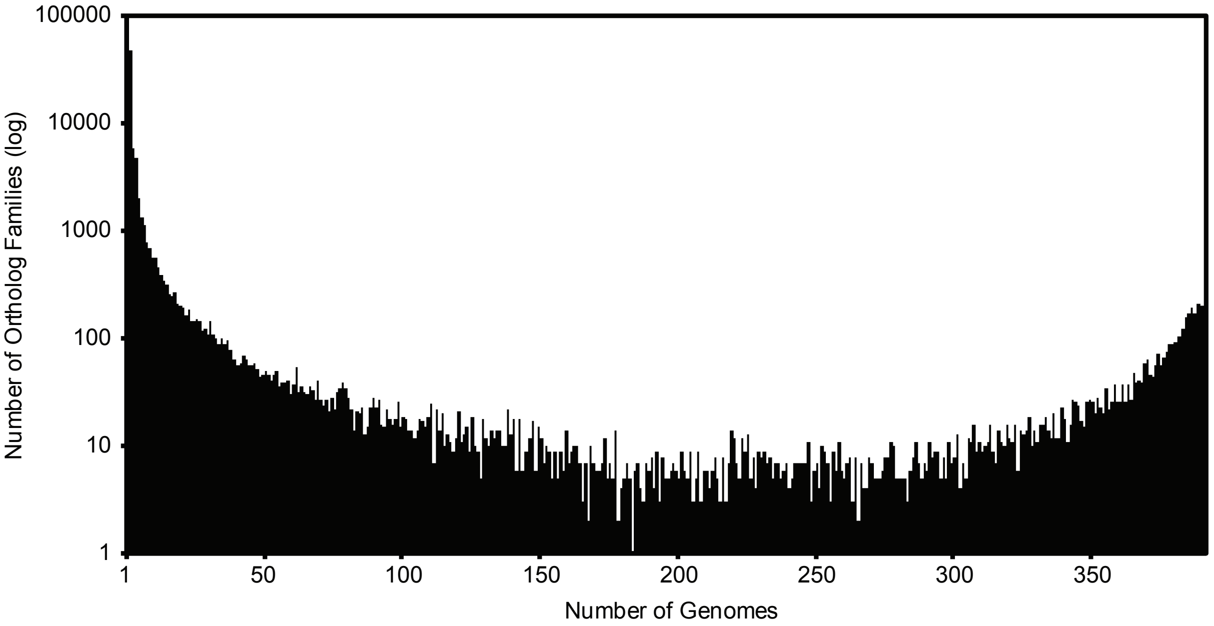

Fig. S5: Number of genomes in which each ortholog family resides. The tendency of ortholog families to exist in very few or nearly all genomes suggests that lateral gene transfer is relatively common in the *Pseudomonas syringae* species complex.

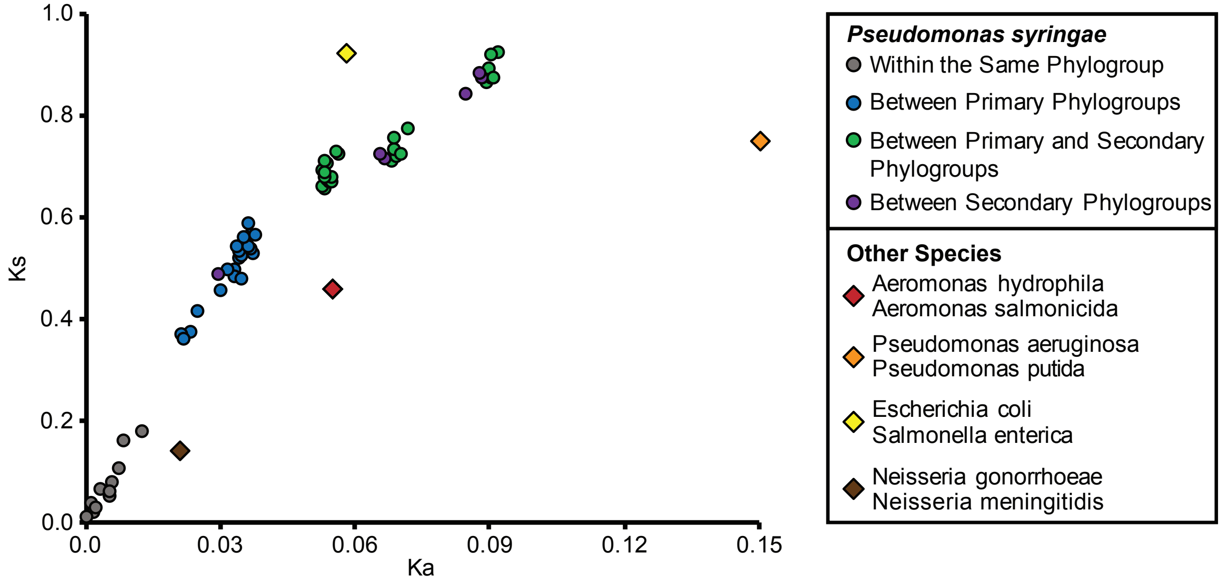

Fig. S6: Evolutionary rates for different strain pairs in the *Pseudomonas syringae* species complex. As expected, non-synonymous (Ka) and synonymous (Ks) substitution rates are lowest in comparisons of *P. syringae* strains within the same phylogroup and highest in comparisons of *P. syringae* strains in primary vs. secondary phylogroups or in different secondary phylogroups. The largest Ka and Ks values observed between strains of the *P. syringae* species complex are comparable to those that we obtained for distinct species comparisons.

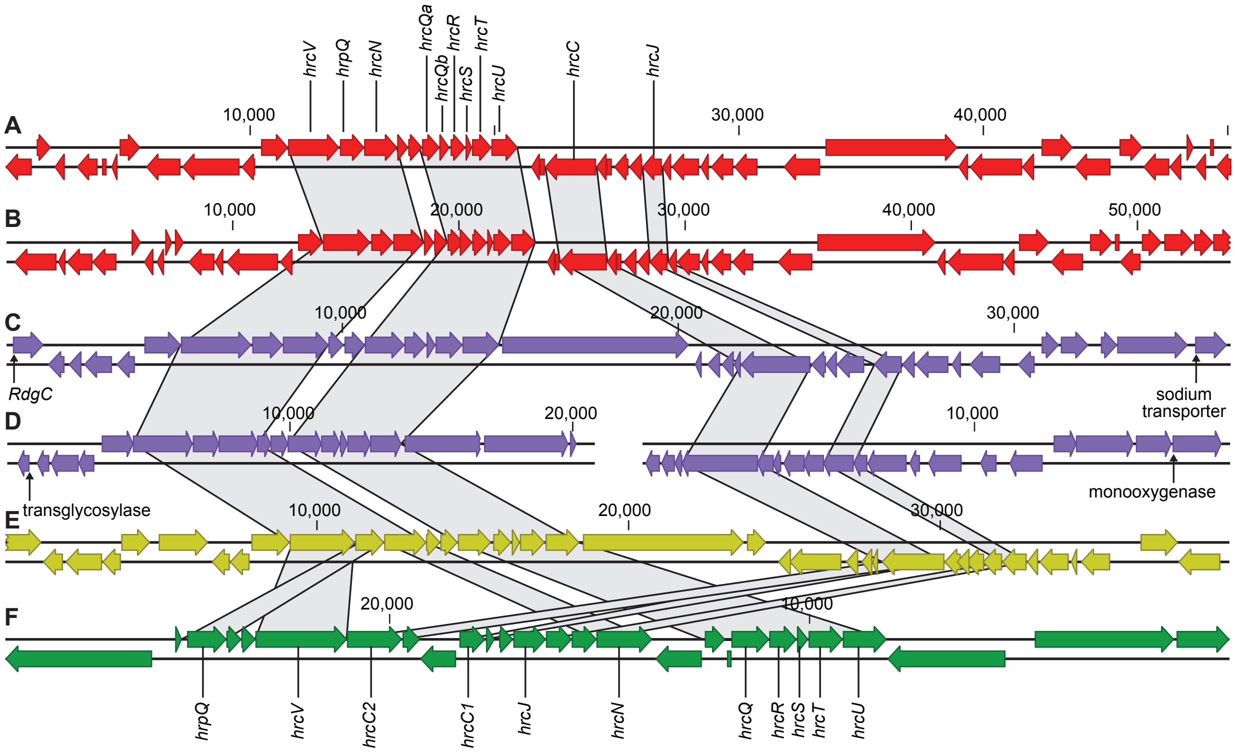

Fig. S7: Genetic architecture of the different type III secretion systems found in the *Pseudomonas syringae* species complex. A) Canonical T-PAI T3SS (*P. syringae* pv. *tomato* DC3000; Genbank AE016853.1). B) Alternate T-PAI T3SS (*P. viridiflava* PNA3.3a; Genbank AY597278). C) Atypical-A A-PAI T3SS (*P. syringae* Psy642; Genbank NZ_ADGB01000014). D) Atypical-B A-PAI T3SS (*P. syringae* PsyUB246; Genbank NZ_KE695357, NZ_KE695399). E) S-PAI T3SS, *P. viridiflava* RMX3.1b; Genbank AY597283). F) *Rhizobial* R-PAI T3SS (*P. syringae* pv. *phaseolicola* 1448a; Genbank CP000058.1). The ten core genes common to all T3SSs in *P. syringae* are labelled on the canonical T-PAI T3SS (A) and the *Rhizobial* R-PAI T3SS (F) and are connected to all other forms of the T3SS with grey boxes.

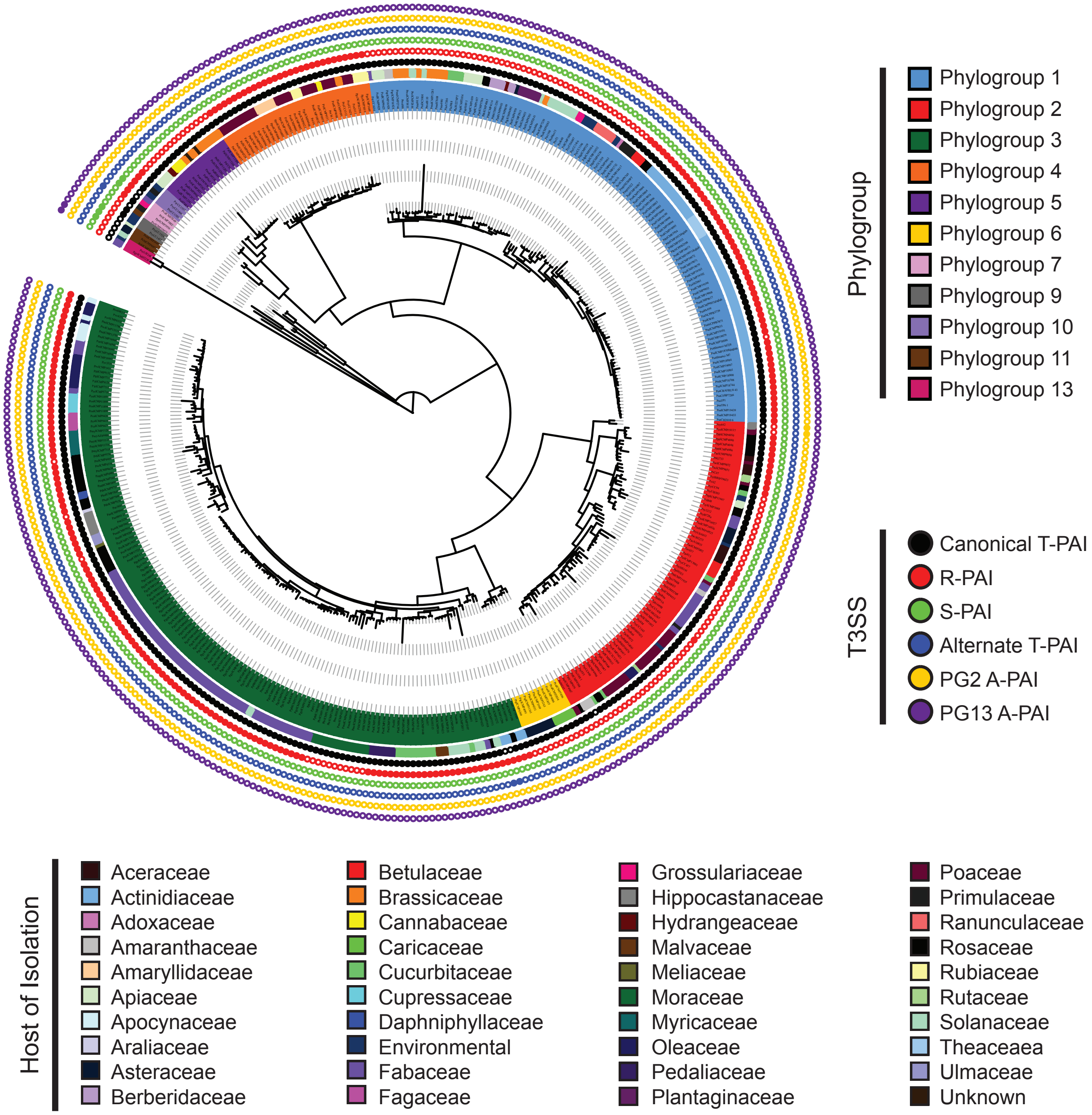

Fig. S8: Distribution of the different *Pseudomonas syringae* complex type III secretion systems on the core-genome phylogenetic tree. All forms of T3SS are mapped to the core genome tree from Fig. 2A, with filled circles representing that the T3SS is present in the strain and empty circles representing that the T3SS is absent in the strain.

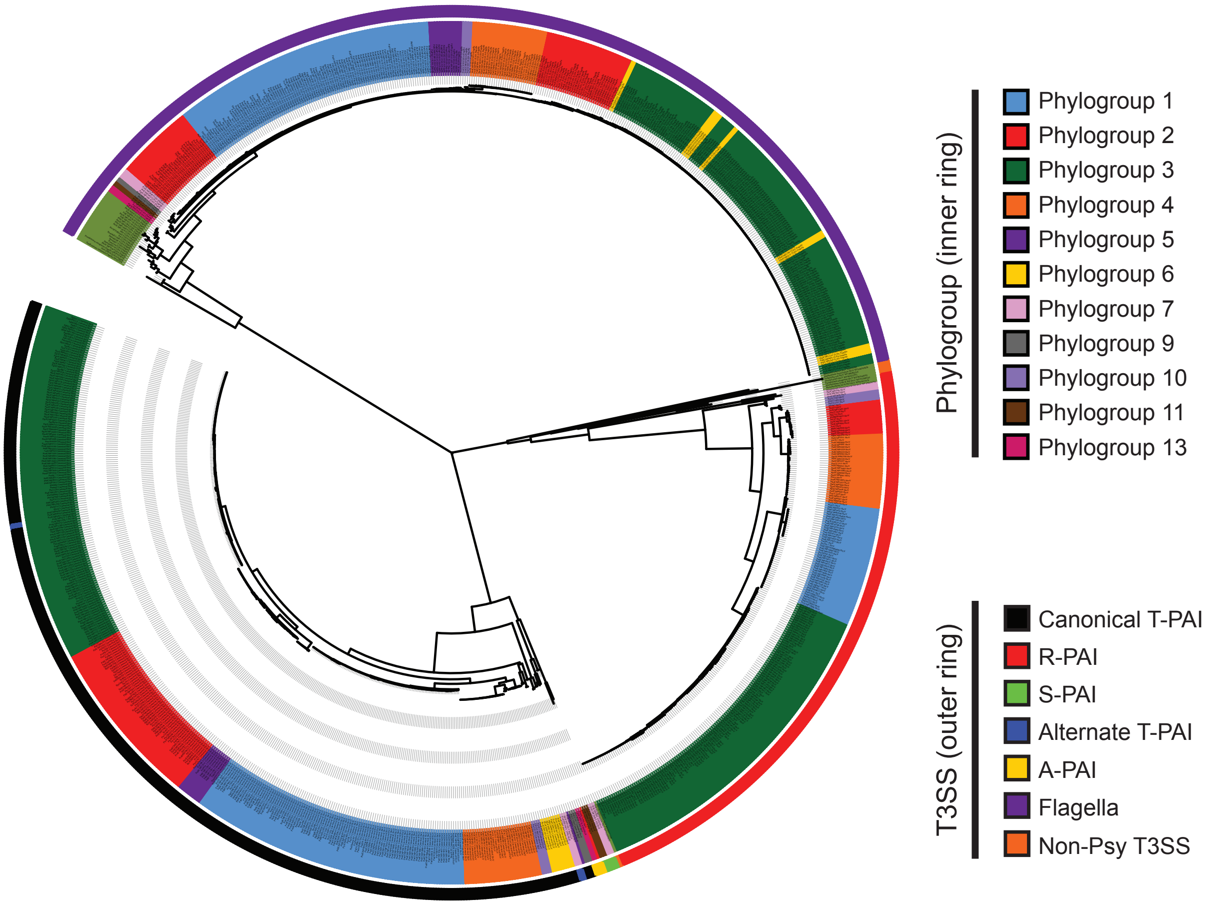

Fig. S9: Maximum likelihood phylogenetic tree of the HrcV structural protein found in all *Pseudomonas syringae* complex type III secretion systems. The HrcV proteins of all T3SSs in all *P. syringae* complex strains and the flagellar HrcV homologs (FlhA) in all *P. syringae* complex strains were aligned using Kalign, along with the HrcV and FlhA homologs from 12 outgroup strains: *Sinorhizobium fredii* (WP_015888343), *Mesorhizobium loti* (WP_010913979), *Pseudomonas fluorescence* (WP_046054793), *Xanthomonas euvesicatoria* (WP_046934829), *Yersinia pestis* (WP_002212971), *Pseudomonas aeruginosa* (WP_003100792), *Salmonella enterica* (EDX52581), *Escherichia coli* (WP_000482518), *Erwinia amylovora* (WP_004155349), *Pantoea agglomerans* (WP_064690655), *Pseudomonas aeruginosa* (FLHA, Q9I3P9_PSEAE), *Xanthomonas campestris* (FLHA, NP_637274). The maximum likelihood tree was constructed with 100 bootstraps using FastTree. Strains are colored based on their *P. syringae* phylogroup, and the outer ring represents the form of T3SS the HrcV/FlhA gene was derived from.

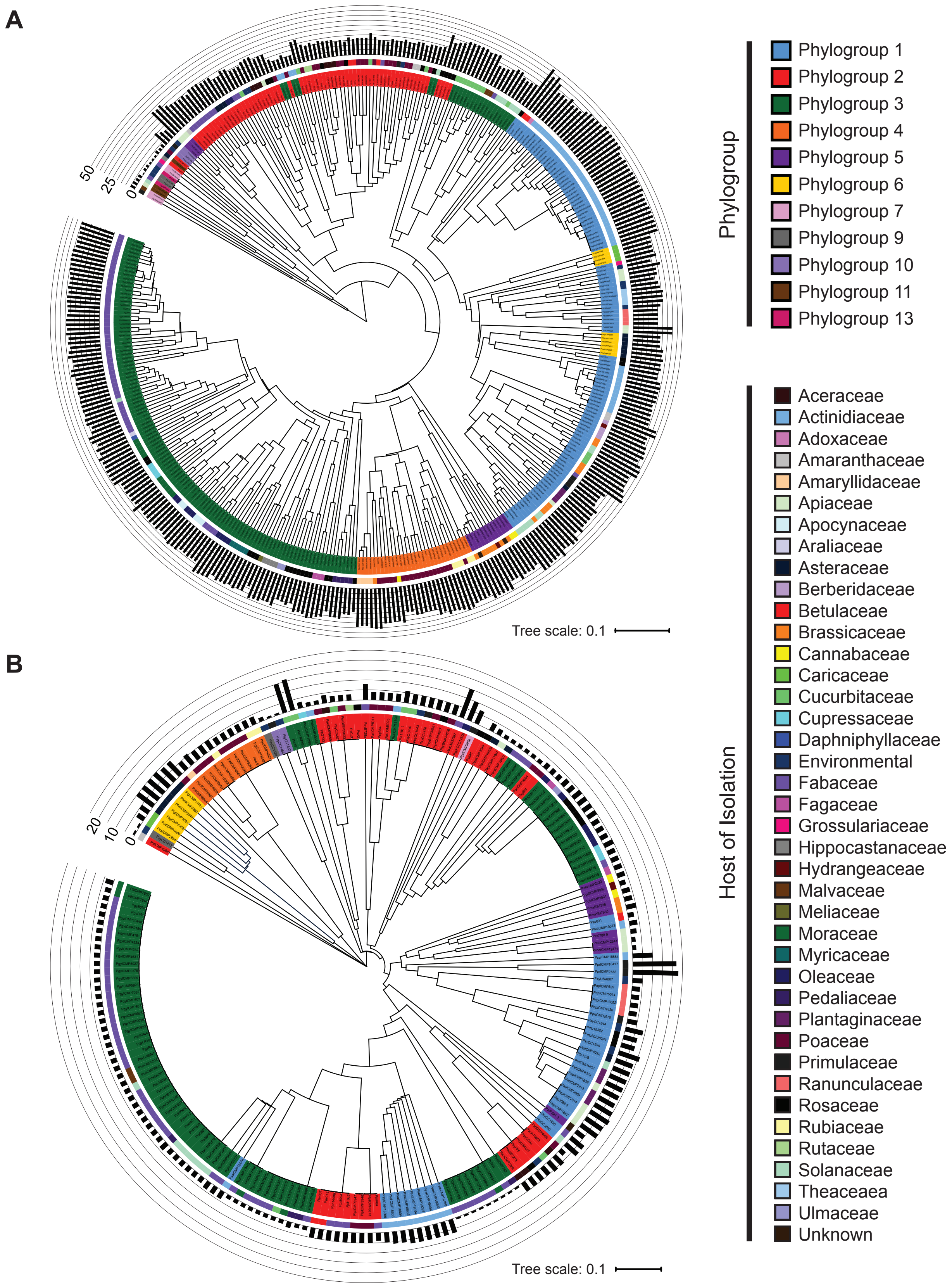
A
B
50
25
0
20
10
0
Tree scale: 0.1
Tree scale: 0.1
Phylogroup
Phylogroup 1
Phylogroup 2
Phylogroup 3
Phylogroup 4
Phylogroup 5
Phylogroup 6
Phylogroup 7
Phylogroup 9
Phylogroup 10
Phylogroup 11
Phylogroup 13
Host of Isolation
Aceraceae
Actinidiaceae
Adoxaceae
Amaranthaceae
Amaryllidaceae
Apiaceae
Apocynaceae
Araliaceae
Asteraceae
Berberidaceae
Betulaceae
Brassicaceae
Cannabaceae
Caricaceae
Cucurbitaceae
Cupressaceae
Daphniphyllaceae
Environmental
Fabaceae
Fagaceae
Grossulariaceae
Hippocastanaceae
Hydrangeaceae
Malvaceae
Meliaceae
Moraceae
Myricaceae
Oleaceae
Pedaliaceae
Plantaginaceae
Poaceae
Primulaceae
Ranunculaceae
Rosaceae
Rubiaceae
Rutaceae
Solanaceae
Theaceaea
Ulmaceae
Unknown

Fig. S10: Phylogenetic analysis of *Pseudomonas syringae* strains based on type III secreted effector (T3SE) content (A) and exchangeable effector locus (EEL) content (B). Both trees were generated by hierarchical clustering of content using the Jaccard coefficient method for calculating the distance between strains and the Ward hierarchical clustering method for clustering. Strain phylogroups, hosts of isolation, and the number T3SE or EEL genes in the corresponding strain are highlighted on each tree.

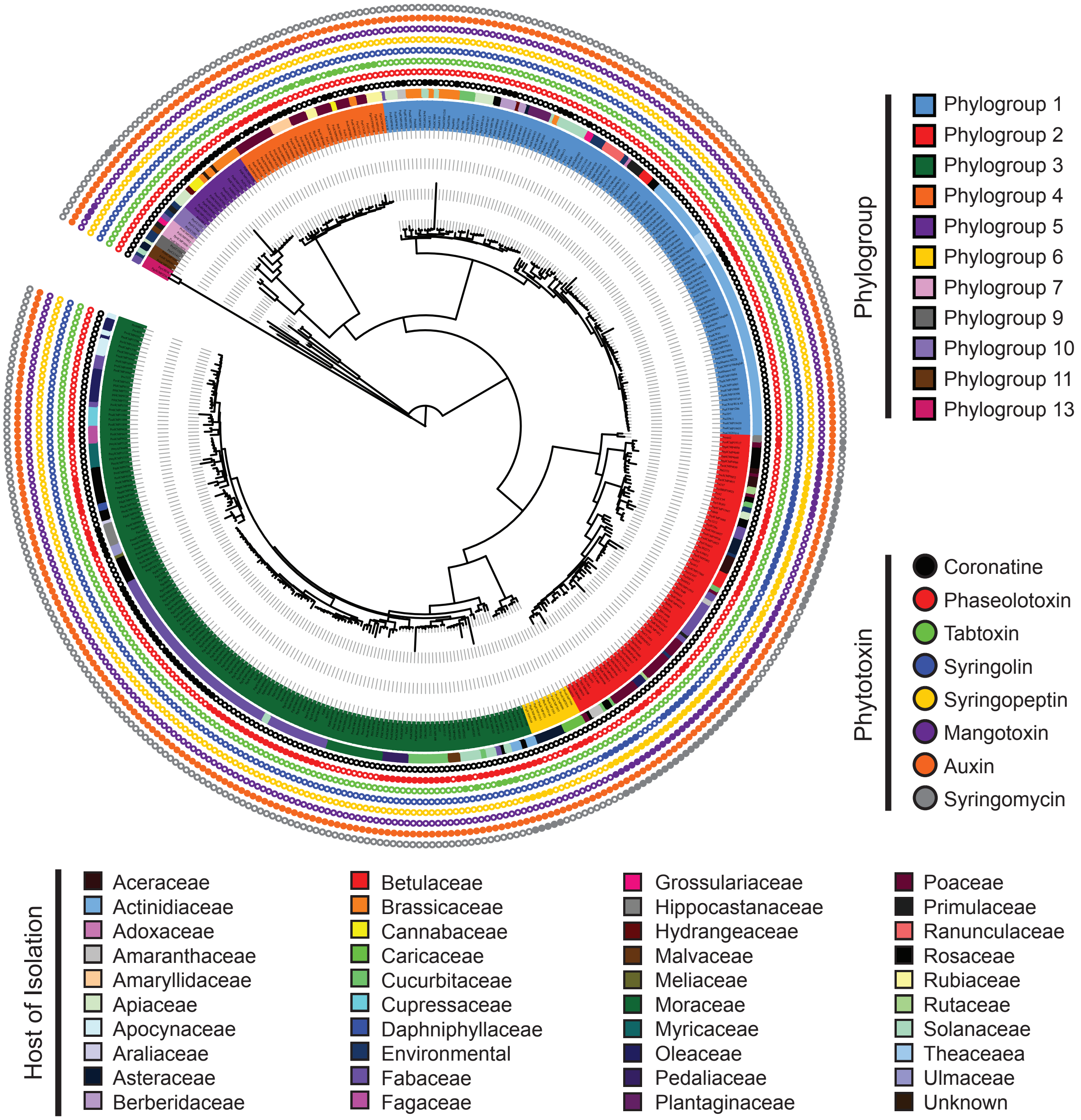

Fig. S11: Phylogenetic distribution of eight major phytotoxins produced by *Pseudomonas syringae* strains. All phytotoxins are mapped to the core genome tree from Fig. 2A, with filled circles representing that the phytotoxin is produced in the strain and empty circles representing that the phytotoxin is not produced in the strain.

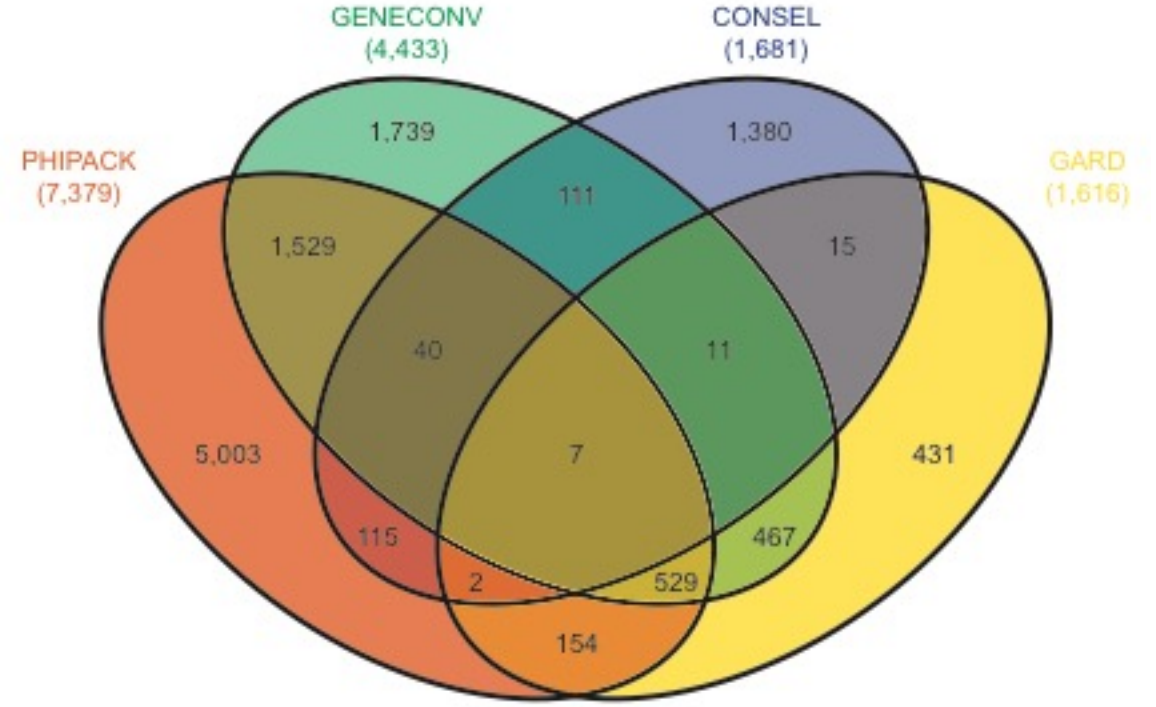

Fig. S12: Comparison of the results of four different recombination analysis pipelines. In analyzing 17,807 ortholog families present in at least five *Pseudomonas syringae* strains, GARD, CONSEL, GENECONV, and PHIPACK identified 1,616, 1,681, 4,433, and 7,379 ortholog families undergoing recombination, respectively, with relatively little overlap between packages. A recombination event was considered significant if the adjusted p-value for the test was less than 0.05 after correcting for multiple comparisons using the Bonferroni correction.

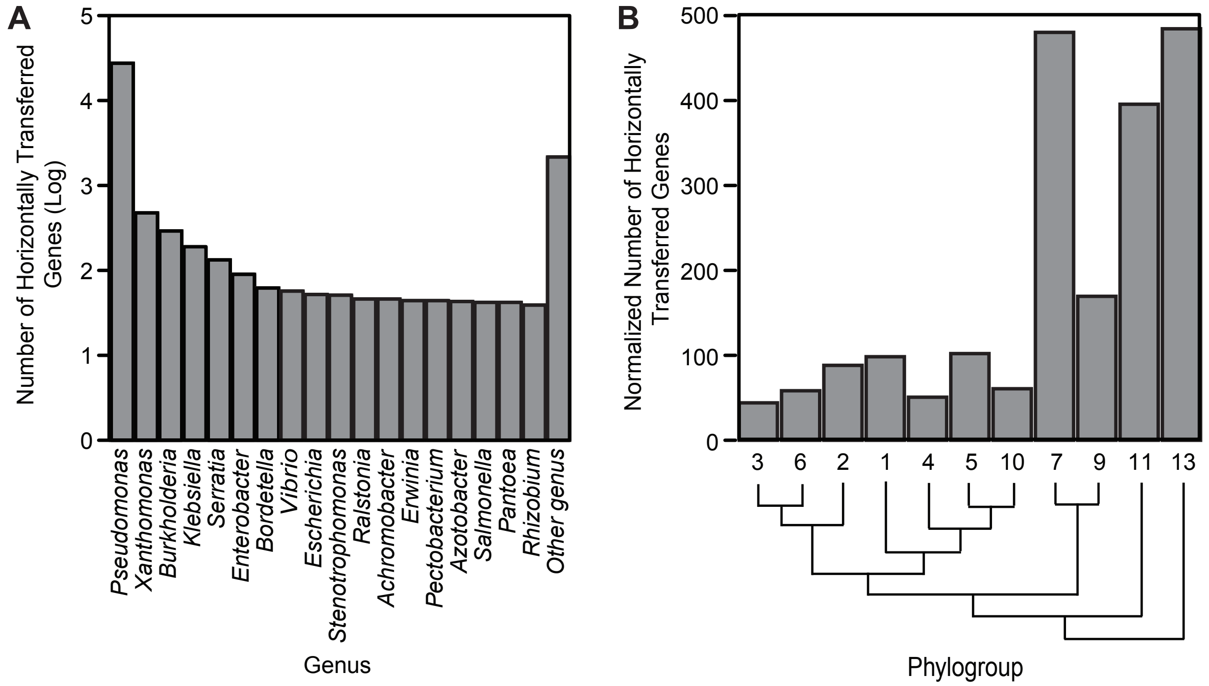

Fig. S13: Frequency of horizontal gene transfer into the *Pseudomonas syringae* species complex. A) Number of putatively horizontally transferred genes contributed by frequent donor genera into *P. syringae* species complex. B) Number of putatively horizontally transferred genes into each phylogroup, normalized by the number of strains in each phylogroup.

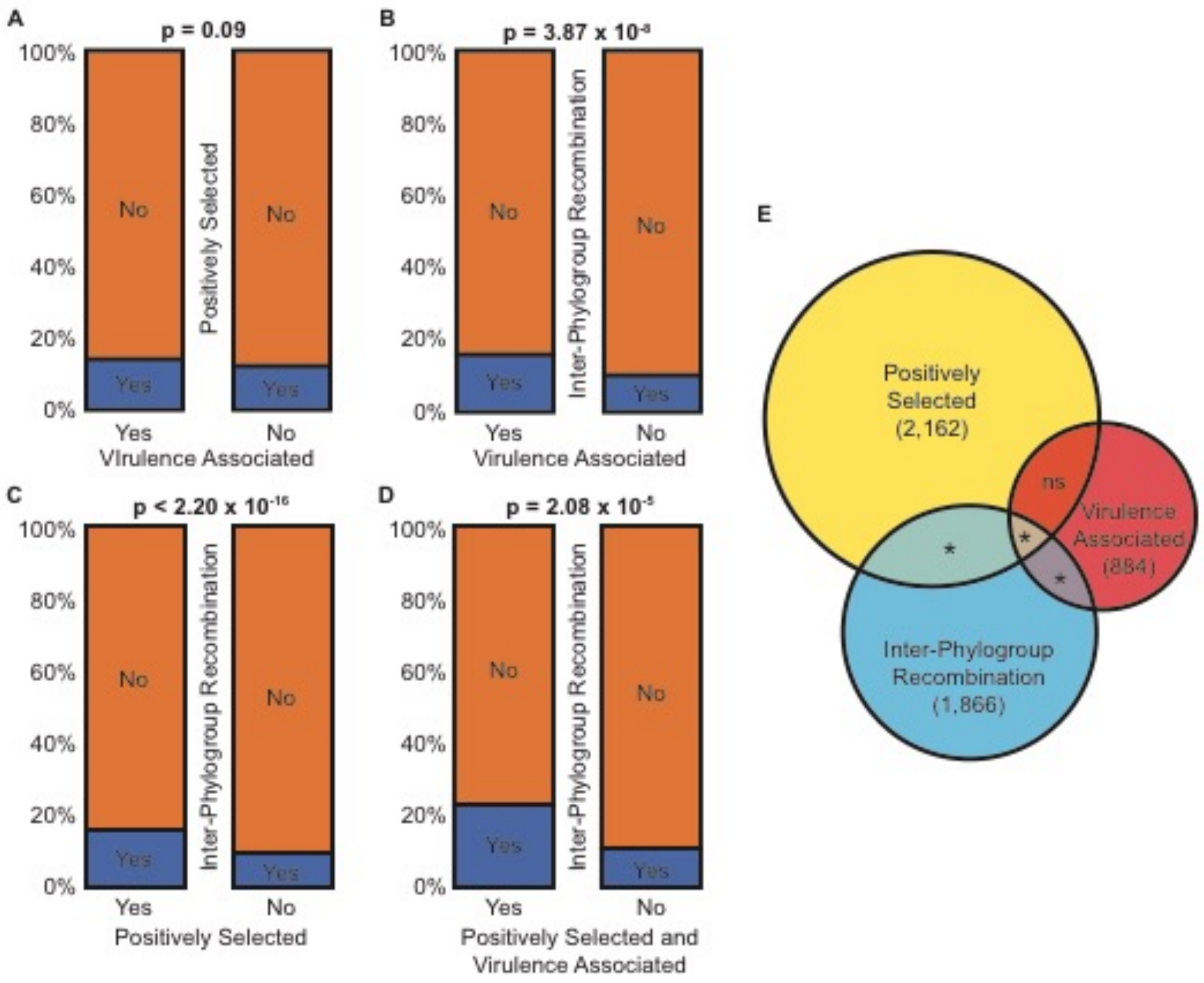

Fig. S14: Relationships between inter-phylogroup recombination, virulence, and positive selection for genes in primary *Pseudomonas syringae* phylogroups based on chi-squared proportions tests. There is no significant association between positively selected and virulence associated genes (A). However, there is a significant positive association between gene families that have undergone inter-phylogroup recombination with virulence-associated gene families (B), positively selected gene families (C), and the small collection of gene families that are both virulence-associated and positively selected (D). The Venn diagram (E) depicts the number primary phylogroup gene families undergoing inter-phylogroup recombination, the number of gene families that are virulence associated, and the number of gene families that are positively selected, as well as the significance of the overlap between these families.

Table S1: Gene Ontology annotations assigned to the novel candidate type III effectors in the *Pseudomonas syringae* species complex.

GO AnnotationTerms	Number of T3SE Candidates
Type III Effector	2765
Type III Secretion Related Protein	2560
Peptidase Activity	46
DNA Binding	25
Regulation of Protein Secretion	24
Regulation of Transcription	20
Metabolic Process	18
Membrane	12
Pathogenesis	11
Cysteine Endopeptidase Activity	9
Oxidation-Reduction Process	7
ATP Binding	4
Transmembrane Transport	4
Uracil Reductase Activity	3
DNA Integration	3
Signal Transduction	3
Extracellular Region	2
Extracellular Space	2
Hydrolase Activity	2
Iron-Sulfur Cluster Binding	2
Methyltransferase Activity	2
Polygalacturonase Activity	2
Transaminase Activity	2
Arginine Metabolic Process	1
Benzoate Transporter Activity	1
DNA Polymerase Activity	1
Flavin Adenine Dinucleotide Binding	1
Heme Binding	1
Outer Membrane	1
Protein Binding	1
Riboflavin Reductase Activity	1
Translation Alongation Factor Activity	1
Unknown Function	727
Total	6264

Table S2: Gene Ontology (GO) terms significantly associated with the virulence related ortholog families in the *Pseudomonas syringae* species complex (FDR p-value < 0.05).

GO ID	GO Term	P-Value (FDR)
GO:0006928	movement of cell or subcellular component	4.63E-15
GO:0001539	cilium or flagellum-dependent cell motility	1.84E-14
GO:0048870	cell motility	1.84E-14
GO:0051674	localization of cell	1.84E-14
GO:0071973	bacterial-type flagellum-dependent cell motility	1.84E-14
GO:0097588	archaeal or bacterial-type flagellum-dependent cell motility	1.84E-14
GO:0009405	pathogenesis	2.23E-12
GO:0040011	locomotion	2.72E-10
GO:0051704	multi-organism process	2.00E-08
GO:0046903	secretion	2.33E-08
GO:0051649	establishment of localization in cell	6.06E-08
GO:0051641	cellular localization	7.14E-08
GO:0015031	protein transport	1.14E-07
GO:0008104	protein localization	1.24E-07
GO:0045184	establishment of protein localization	1.24E-07
GO:0009306	protein secretion	1.55E-07
GO:0032940	secretion by cell	1.55E-07
GO:0033036	macromolecule localization	3.02E-07
GO:0032879	regulation of localization	1.24E-04
GO:0032880	regulation of protein localization	1.24E-04
GO:0050708	regulation of protein secretion	1.24E-04
GO:0051046	regulation of secretion	1.24E-04
GO:0051049	regulation of transport	1.24E-04
GO:0051223	regulation of protein transport	1.24E-04
GO:0060341	regulation of cellular localization	1.24E-04
GO:0070201	regulation of establishment of protein localization	1.24E-04
GO:1903530	regulation of secretion by cell	1.24E-04
GO:0009237	siderophore metabolic process	1.61E-03
GO:0019290	siderophore biosynthetic process	1.61E-03
GO:0044550	secondary metabolite biosynthetic process	6.84E-03
GO:0044781	bacterial-type flagellum organization	6.84E-03
GO:1902589	single-organism organelle organization	6.84E-03
GO:0008152	metabolic process	2.47E-02
GO:0006471	protein ADP-ribosylation	3.73E-02
GO:0019184	nonribosomal peptide biosynthetic process	3.73E-02
GO:0030030	cell projection organization	3.73E-02

Table S3: Results of chi-squared equality of proportions tests for relationships between inter-phylogroup recombination, virulence, and positive selection in gene families from primary *Pseudomonas syringae* phylogroups.

Gene Group A	Gene Group B	χ^2	df	p
Ecologically Significant	Evolutionarily Significant	2.82	1	0.0931
Ecologically Significant	Inter-Phylogroup Recombination	30.21	1	<0.0001
Evolutionarily Significant	Inter-Phylogroup Recombination	115.05	1	<0.0001
Eco. and Evo. Significant	Inter-Phylogroup Recombination	18.11	1	<0.0001
